# The psychosocial determinants of quality of life in breast cancer survivors: a scoping review

**DOI:** 10.1186/s12885-020-07389-w

**Published:** 2020-10-02

**Authors:** Michael G. Culbertson, Kathleen Bennett, Catherine M. Kelly, Linda Sharp, Caitriona Cahir

**Affiliations:** 1 Division of Population Health Sciences, Royal College of Surgeons in Ireland, Beaux Lane House, Mercer Street, Dublin, 2 Ireland; 2 Mater Misericordiae University Hospital, Dublin, 1 Ireland; 3 Population Health Sciences Institute, Newcastle University Centre for Cancer, Newcastle University, Newcastle, UK

**Keywords:** Breast cancer, Survivorship, Quality of life, Psychosocial, Scoping review

## Abstract

**Background:**

Breast cancer care today involves state-of-the-art biomedical treatment but can fail to address the broader psychosocial and quality-of-life (QoL) issues associated with the transition to breast cancer survivorship. This scoping review examines the evidence on the influence of psychosocial determinants on QoL in breast cancer survivors.

**Methods:**

Scoping review methodology was used to: (1) identify the research question(s); (2) identify relevant studies; (3) undertake study selection; (4) extract data; (5) collate, summarise and report the results.

**Results:**

A total of 33 studies met the inclusion criteria. The majority of studies were conducted in the US (*n* = 22, 67%) and were mainly cross-sectional (*n* = 26, 79%). Sixteen psychosocial determinants of QoL were identified. Social support (*n* = 14, 42%), depression (*n* = 7, 21%) and future appraisal and perspective (*n* = 7, 21%) were the most frequently investigated determinants. Twelve different QoL measures were used. A range of different measurement tools were also used per psychosocial determinant (weighted average = 6). The 14 studies that measured the influence of social support on QoL employed 10 different measures of social support and 7 different measures of QoL. In general, across all 33 studies, a higher level of a positive influence and a lower level of a negative influence of a psychosocial determinant was associated with a better QoL e.g. higher social support and lower levels of depression were associated with a higher/better QoL. For some determinants such as spirituality and coping skills the influence on QoL varied, but these determinants were less commonly investigated.

**Conclusion:**

Consensus around measures of QoL and psychological determinants would be valuable and would enable research to determine the influence of psychosocial determinants on QoL adequately. Research in other healthcare settings beyond the US is required, in order to understand the influence of organisation and follow-up clinical and supportive care on psychosocial determinants and QoL and to improve the quality of care in breast cancer survivors.

## Background

In recent years, with earlier diagnosis and better treatment options, breast cancer survival in women has increased steadily and 5-year net survival in high income countries is now 85–90% [1]. This means that millions of women worldwide are now living with, and beyond, a breast cancer diagnosis; the 5 year prevalence of breast cancer is approximately seven million globally [2, 3]. The concept of breast cancer survivorship encompasses the wider physical, psychological, social and economic issues of breast cancer [4, 5]. The transition from breast cancer patient to breast cancer survivorship brings numerous uncertainties for women [6]. The end of hospital-focused cancer treatment typically includes the loss of the safety net of active medical treatment, a resumption or alteration of former roles within and outside the home, a decline in interpersonal support and ongoing physical and psychological effects of diagnosis and treatment, such as fatigue, sleep disturbance, sexual dysfunction, urinary/bowel problems, and cognitive problems [7, 8]. However, while breast cancer care today often provides state-of-the-art biomedical treatment, it can fail to address the broader psychosocial and quality-of-life (QoL) issues associated with survivorship [9].

Psychosocial factors have been defined as any exposure that may influence a physical health outcome through a psychological mechanism [10]. Psychosocial factors can include depression and other emotional problems, psychological traits and disruptions in the social environment, all of which can compromise the effectiveness of health care and adversely impact breast cancer survivors’ return to good health [9]. Major depression, for example, is substantially more common in people with cancer than the general population and mostly goes untreated in the outpatient setting [11]. There is some evidence that psychosocial factors are associated with impairments in QoL in breast cancer survivors [8]. High social isolation and lack of social support have been reported to be associated with a lower QoL in breast cancer survivors [12]. In contrast, personality attributes such as optimism (i.e. general expectancy for positive outcomes) and use of active coping strategies such as problem solving, identifying benefits in the experience and expressing cancer-related emotions are all associated with greater psychological adjustment and an improved QoL [13].

Some studies have indicated that breast cancer survivors have a significantly lower QoL, including lower physical, functional, emotional and social well-being compared to control-matched healthy populations and experience clinically relevant restrictions in several QoL dimensions 10 years after diagnosis, with restrictions in role, cognitive and social functioning and fatigue increasing over time [12, 14]. While other studies have found that 10 years after diagnosis, many women report having a new meaning to their lives and healthier lifestyles, with long-term survivors having similar or improved QoL levels when compared to age-matched controls who have never had breast cancer [15, 16]. To inform survivorship care planning, it would be valuable to better understand which psychosocial factors are associated with improved or worsened QoL in breast cancer survivorship. Such an understanding would inform evidence-based psychosocial care and enable the development of targeted interventions to enhance QoL and reduce long term psychological and physical morbidity [6, 17]. This scoping review, therefore, examines the evidence on the influence of psychosocial determinants on QoL in breast cancer survivors.

## Methods

This scoping review seeks to identify the current literature published in this field, examine how the research was conducted and identify the key factors related to this topic and gaps in knowledge [18]. The scoping review framework of Arksey and O’Malley [19] and later advanced by Levac, Colquhoun [20] was used to guide the current study. This framework includes five stages: (1) identifying the research question(s); (2) identifying relevant studies; (3) study selection; (4) data extraction; (5) collation, summarising and reporting the results [19].

### Identifying the research question

This scoping review was developed to describe the nature, number and scope of published research articles measuring the association between psychosocial determinants and QoL in breast cancer survivors.

### Identifying relevant studies

A systematic literature search of the databases, PubMed, Embase, PsycINFO, and CINAHL was conducted of all articles published between 01/01/1998 and 31/12/2018. The electronic search strategy included MeSH headings, key words and their derivatives “breast cancer, survivor, quality of life” (Appendix). The terms and the search criteria were developed and tested with a medical librarian. All articles were downloaded into Endnote and duplicates were removed.

### Study selection

The titles and abstracts of all identified studies were screened by an independent team of reviewers. One reviewer independently applied the inclusion criteria (Table 1) to each abstract and a random sample of 75% of the abstracts were reviewed independently by a second reviewer. The review team met to compare screened abstracts and any differences were resolved through consultation with a third reviewer.

**Table 1:** Study inclusion and exclusion criteria

Study characteristics	Inclusion criteria	Exclusion criteria
**Abstract Criteria**		
Participants	Women Aged 18+ Breast cancer survivor (post-treatment)	Initial diagnosis of breast cancer/pre-cancer treatment
Study Design	Observational studies e.g. retrospective or prospective cohort studies, cross-sectional studies	Systematic reviews, randomised controlled trials of intervention or treatment studies
Outcome Measure- quality of life (QoL)	Overall QoL Physical well-being Emotional well-being Functional well-being Social well-being	QoL not reported in the abstract
Psychosocial Determinants	Psychosocial determinants	Clinical, treatment, or socio-demographic determinants only reported in the abstract
Publication	Peer-Reviewed Journal Published in -the last 20 years -in English	Doctoral Dissertation Conference proceeding e.g. abstract, poster
**Full Text Criteria**		
Population	Women Aged 18+ Stage I-III breast cancer (non-metastatic) Completed breast cancer treatment Breast cancer survivor (post-treatment)	Initial diagnosis of breast cancer/pre-cancer treatment Metastatic breast cancer or Ductal Carcinoma in situ Currently receiving breast cancer treatment (e.g. chemotherapy, radiotherapy, excluding endocrine therapy) Participants of a clinical trial
Outcome Measure (QoL)	Validated QoL measure Generic and specific to breast cancer Overall/global QoL	Non-validated QoL measure (developed by authors) Aspects of QoL e.g. emotional well-being, depression
Psychosocial Determinants	At least one modifiable psychosocial determinants, e.g. depression, social support	Clinical, treatment, or socio-demographic determinants only Non-modifiable behavioural determinants only
Publication	Peer-Reviewed Journal Published in -the last 20 years -in English	Doctoral Dissertation Conference proceeding abstract or poster

The inclusion criteria were then refined and a more detailed set of criteria was developed for the full text review process (Table 1). The breast cancer survivorship definition was refined to only include women who had completed their hospital-focused breast cancer treatment e.g., women had to be post-surgery, chemotherapy and radiotherapy treatments (if applicable). The criteria for the QoL measure was refined to include only validated measures of overall QoL (e.g. FACT-B, EORTC QLQ-C30) [21, 22]. At least one of the psychosocial determinants measured had to be potentially modifiable (e.g. depression, social support). Two reviewers independently reviewed the full texts of all the identified abstracts using these more detailed inclusion criteria. The reference lists of eligible studies were also reviewed to identify any further studies that had been missed in the electronic searches.

### Data extraction

After reading the full-texts of each study to be included in the review, two researchers independently extracted the following data: author(s), year of publication, study design, study location, participant characteristics, time period, psychosocial determinant(s) and how they were measured, QoL outcome and how it was measured and the main findings and any adjustments for covariates. Data was initially extracted from the first 5 studies and compared by the two reviewers to ensure consistency.

### Collating and summarising the data

The data from the included studies was collated by both psychosocial determinants and QoL measures to provide both a descriptive and numerical summary of the findings and to answer the following four research questions;

1. What are the main psychosocial determinants of QoL in breast cancer survivors that have been investigated to date?
2. What are the most frequently used measurement tools to assess QoL in breast cancer survivors?
3. Which psychosocial determinants measurement tools were used and how frequently were they used per individual QoL measures?
4. What is the influence of these psychosocial determinants on QoL in breast cancer survivors?

## Results

### Study population

**Fig. 1:**
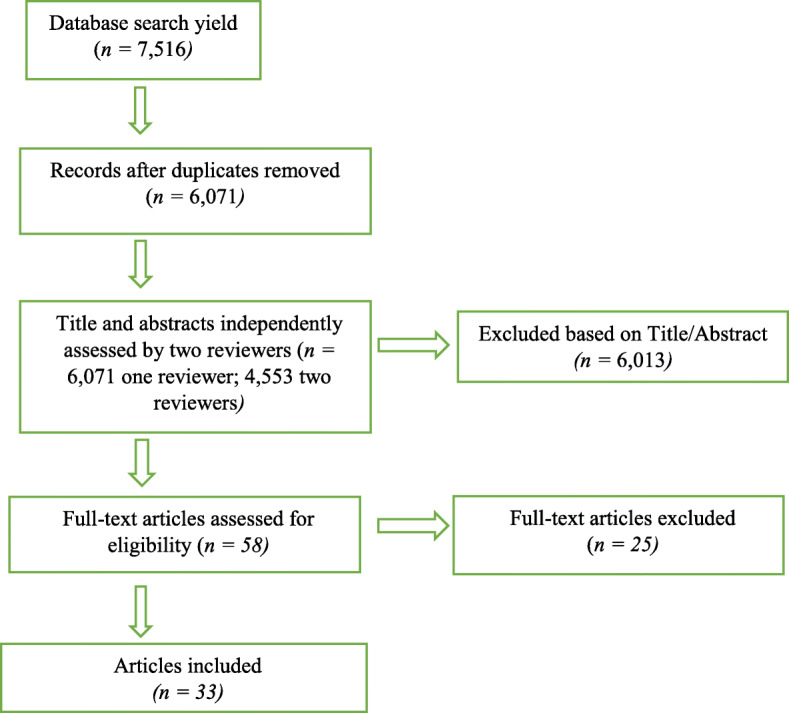
PRISMA diagram of selection of studies

The study selection process is outline in Fig. 1. The four databases yielded 7516 citations, which reduced to 6071 after removing duplicates. Of these, 58 full-texts were deemed potentially eligible and reviewed in full text. Of these, 33 studies were eligible for inclusion in this scoping review.

The majority of included studies (*n* = 26, 79%) employed a cross-sectional design; the remaining 7 studies (21%) assessed and compared QoL at various different time points post diagnosis e.g. short term at 6, 12 and 18 months [23, 24], medium term 2–4 years [25–27] and longer term 5–13 years later [13, 28]. Most studies were conducted in the United States (*n* = 22, 67%), with the remainder from China (*n* = 3, 9%), Germany (*n* = 2, 6%), and single studies in Australia, Taiwan, Malaysia, Japan, Korea and Austria respectively. Sample size ranged from 51 to 2671 participants; the average was 418 [29, 30]. The average age of participants was mid-40s to mid-50s, but ranged from 18 years to 96 years. The time period since completing breast cancer treatment varied greatly; some studies assessed the psychosocial determinants of QoL 6 months to 1 year post-treatment [28, 31], while other studies included participants who completed treatment up to 35 years previously [32].

### Identifying psychosocial determinants of QoL in breast cancer survivors

The eligible studies reported on 16 possible psychosocial determinants of QoL (Table 2). The most prevalent psychosocial determinants investigated were social support (investigated in 14 studies), depression (7 studies) and future appraisal and perspective (7 studies). Five studies assessed coping, optimism and stress determinants and 4 studies assessed spirituality. Three studies looked at anxiety, confidence and self-efficacy and impact of events. Two studies investigated post-traumatic growth and there were single studies for positive and negative affect, cognitive symptoms, work limitations and health care system factors.

**Table 2:** Psychosocial determinants of QoL in breast cancer survivors

Primary Author, Year	Study design	Country	Participant characteristics	Cancer stage & treatment	Time period	Psychosocial measure (predictor)	Quality of Life measure (outcome)	Results	Covariates (adjusted)
***Social Support***									
Ashing –Giwa K.T.. 2010 [33]	Cross-sectional	United States	703 participants aged 29–91 years (mean = 55, SD = 13). European- (*n* = 179), African- (*n* = 135), Latina- (*n* = 183), and Asian- (*n* = 206) Americans. 14.4% = < secondary education; 10.8% = completed secondary education; 74.8% = > secondary education	11.1% = Stage 0 36.7% = Stage 1 38.5% = Stage 2 13.7% = Stage 3 58.5% = Lumpectomy/other; 38.4% = Mastectomy; 15.6% = Mastectomy and reconstruction; 57.8% = Chemotherapy; 66.0% = Radiation	1–5 years since diagnosis (mean = 3 years)	Medical Outcomes Study (MOS) Social Support Survey	FACT-B- Physical and emotional well-being scale SF-36 -general health perception sub-domain and pain sub-domain	Social support did not have a significant direct relationship with QoL	Life stress scale. Health care system- patient-doctor relationship, comfort in health care system and diagnostic care delay and sociodemographic variables
Carver C.S. 2006 [13]	Longitudinal	United States	163 women with a mean age of 51.4 (SD = 10.61). 70% = Caucasian, 20% = Hispanic; 10% = African American 72% were married	3% = Stage 0 62% = Stage 1 35% = Stage 2 53% = Lumpectomy 47% = Mastectomy 31% = Chemotherapy 50% = Radiation 56% = Tamoxifen 25% = Reconstruction	Recruitment between 1988 and 1995 and 1994–1996 Data collection in 2001. 5–13 years since surgery	Interpersonal Support Evaluation List (ISEL)	QLACS	Social support was negatively correlated with lack of positive feelings, pain, sexual impairment, family distress and recurrence distress (subset of 101 participants). Not investigated in multivariate analysis	Optimism, cancer confidence and sociodemographic, clinical and treatment variables
Cheng H, 2013 [34]	Cross-sectional	China	100 Asian/Chinese women aged 37–71 (mean = 53.75, SD = 7.27). 7% = < secondary education; 76% = completed secondary education; 17% = > secondary education 84% were married	15% = Stage 1 61% = Stage 2 24% = Stage 3 44% = Radiotherapy 75% = Hormonal therapy 60% = Traditional Chinese Medicine	Median number of months since treatment was 44 (IQR = 23–61)	Social Support Questionnaire (SSQ-6)	QOL-CS	Participants who had moderate and high levels of social support satisfaction had a significantly better overall QOL as well as better physical psychological and social QoL	Annual household income and length of survivorship
DiSipio T et al., 2009 [35]	Cross-sectional	Australia	323 women. 67% of women were aged ≥50 years. 202 regional based and 121 rural 54% = < secondary education; 32% = completed secondary education; 14% = > secondary education 77% were married	61% = Complete local excision 39% = Mastectomy / partial / radical Adjuvant treatment 18% = No treatment 82% = Chemotherapy / Radiotherapy	Recruitment between April 2006 March 2012 post diagnosis	Social Networks Index Supportive Care Needs Survey – Health system and information needs	FACT-B	Lack of a confidante was associated with a significantly lower QoL Lower health care service needs was associated with a higher QoL	Amount of stress, perceived handling of stress, overall health self-efficacy and sociodemographic and clinical variables
Dura-Ferrandis E, 2016 [28]	Longitudinal	United States	1280 women aged 65–91 (mean = 77, SD = 9). 88.1% = Caucasian; 11.9% = Non-Caucasian 42.1% = < or completed secondary education; 57.9% = > secondary education 55.3% were married	45.6% = Stage 1 31.2% = Stage 2a 23.2% = Stage 2b or higher 67.6% = Breast Cancer Surgery 32.4% = Mastectomy 57.0% = Hormonal therapy only 43.0% = Chemotherapy	Recruitment was conducted from January, 2004 -April 2011 with follow-up in June 2011. Baseline data was collected nearly 2 months after last surgery. Follow-up data was collected 6 and 12 months after the baseline interview and annually for up to 7 years	Medical Outcomes Study (MOS) Social Support Survey	EORTC QLQ-C30	Higher tangible support decreased the probability of being in accelerated emotional and physical functional decline group versus maintained high emotional and physical functional group	Optimism, coping and sociodemographic variables
Goyal N. G, 2018 [23]	Longitudinal	United States	565 women aged 25–96 (mean = 55, SD = 16). 90% Caucasian; 10% = Non-Caucasian. 63% were college educated 72% were partnered	52% = Stage 1 40% = Stage 2 8% = Stage 3 36% = Mastectomy 67% = Chemotherapy 72% = Radiation 73% = Hormonal therapy	Baseline data was collected within 8 months of diagnosis. Follow-up data was collected at 6, 12, and 18 months post baseline	Medical Outcomes Study (MOS) Social Support Survey	FACT-B	Those in the “consistently high” QoL trajectory had greater social support compared to all other groups	Depression, coping, spirituality, optimism, Illness Intrusiveness Rating Scale and sociodemographic and clinical variables
Huang C.Y. 2013 [36]	Cross-sectional	Taiwan	150 women aged 23–83 (mean = 56, SD = 10.4). 57% had less than 9 years of education. 77% were married.	13% = Stage 0 27% = Stage 1 46% = Stage 2 75% = Adjuvant treatment	Average duration of treatment was 33 months	Interpersonal Support Evaluation List (ISEL)	SF-36	Appraisal support, self-esteem support and belonging support were significantly associated with physical QoL Belonging support was also significantly associated with mental QoL	Sociodemographic and clinical variables
Janz N.K., 2014 [26]	Longitudinal	United States	772 women aged on average 59.1 (SD = 13). 47.3% = Caucasian; 16.8% = Black; 36.9% = Latina. 21% = < secondary education; 20.9% = completed secondary education; 58.1% = > secondary education 55% were married	55.3% = Stage 1 36.0% = Stage 2 8.7% = Stage 3 40.7% = Mastectomy 57.8% = Lumpectomy 64.5% = Radiation 45.2% = Chemotherapy	Data was taken 9 months post diagnosis. Follow-up occurred 4 years post diagnosis	Emotional support from others and satisfaction with partner scale	FACT-B- Emotional well-being subscale	No association between social support and satisfaction with partner relationship and emotional well-being	Depression, spirituality, appraisal and sociodemographic and clinical variables
Lewis, J., 2001 [37]	Cross-sectional	United States	64 women aged between 30 and 81 years (mean = 59.2 SD = 9.8). 80% = Caucasian; 20% = African American / Hispanic / Asian / West Indian 6.5% = < secondary education 28% = completed secondary education; 65.5% = > secondary education 66% were married	89% = Chemotherapy / Radiation 71% = Mastectomy 23% = Lumpectomy	Last treatment ranged from 1 to 15 years prior (mean = 7)	Interpersonal Support Evaluation List (ISEL) – Appraisal subscale	SF-36	Perceived social support was not associated with physical quality of life but was significantly associated with a better mental quality of life	Impact of Events(Intrusive thoughts) and sociodemographic variables
Sammarco, A., 2008 [32]	Cross-sectional	United States	89 Latina breast cancer survivors with a mean age of 57.35 years (SD = 12.74, range 30–86 years). 65% = Caucasian; 35% = Latina.7% = < secondary education; 41% = completed secondary education; 52% = > secondary education 61% were married	17% = Surgery only 6% = Adjuvant only 77% = Both	Breast cancer treatment was completed between 1 and 35 years prior (mean = 4.99 years, SD = 4.73)	Social Support Questionnaire (SSQ)	QLI-CV	Increased perceived social support was associated with improved QoL	Uncertainty in illness
Northouse, L.L (1999) [38]	Cross-sectional	United States	98 African American women aged 29–81 years (mean = 55, SD = 18). Average education was 13 years (SD = 6). 41% were married	70% = Mastectomy	The average time since diagnosis was 6 years (SD = 3). Time since diagnosis ranged from 1 to 15 years	Family APGAR-family functioning	FACT-B	Family functioning was significantly associated with QoL	Optimism, symptom distress, current concerns, appraisal of illness and sociodemographic and clinical variables
Pedro L.W. (2001) [39]	Cross-sectional	United States	62 women aged ≥60 years. Majority were married, retired, white and college-educated	Majority surgery or a combination of surgery and radiation	5 to 10 years beyond initial diagnosis and disease and recurrence free	Norbeck Social Support Questionnaire (NSSQ)	QLI-CV	A statistically significant univariate inverse relation was found between total loss (recent loss, number of individuals lost, and amount of that loss) and QoL. In multivariate analysis, this relationship was no longer significant	Self-esteem and learned resourcefulness
Edib Z (2016) [40]	Cross-sectional	Malaysia	117 women.13.7% = < 40; 24.8% = 40–49; 61.6% = > 50. 58.1% = Malaysian; 29.9% = Chinese; 12.0% = Indian 29.1% = < secondary education; 39.3% = completed secondary education; 31.6% = > secondary education 77.8% were married	6.8% = Stage 0 20.5% = Stage 1 36.8% = Stage 2 23.9% = Stage 3 12.0% = Stage 4 31.6% = Breast Cancer Surgery 68.4% = Mastectomy 80.3% = Radiotherapy 71.8% = Chemotherapy 79.3% = Hormone therapy 22.6% = Immune therapy	Women were at least 1 year post diagnosis. 42.7% were < 2 years post- diagnosis. 42.7% were 2–5 years post diagnosis and 14.6% were > 5 years post diagnosis	Supportive Care Needs Survey- Short Form	EORTC QLQ-C30	Physical and psychological unmet needs were significantly independently associated with QOL	Sociodemographic and clinical variables
Avis N.E. (2005) [41]	Cross-sectional	United States	202 women between the age of 25 and 50 years (mean 43.5 years). 96% were White. 20.3% = < or completed secondary education; 79.7% = > secondary education 81% were married/partner	43.4% = Mastectomy 75.1% = Chemotherapy 69.6% = Radiation therapy	Diagnosed with their first breast cancer in the previous 3 years and were at least 4 months after diagnosis	Cancer Rehabilitation Evaluation System (CARES)	FACT-B Ladder of Life	Relationship problems was negatively associated with FACT-B and overall QoL (Ladder of Life)	Coping, sociodemographic and clinical variables
***Depression***									
Begovic-Juhant, A., 2012 [42]	Cross-sectional	United States	70 women aged 23–79 (mean = 49.72, SD = 10.62). 65% = Caucasian; 45% = African American.8% = < secondary education; 41% = completed secondary education; 51% > secondary education. 84% reported employment	60% = Mastectomy 27% = Lumpectomy 73% = Chemotherapy 57% = Radiation 36% = Hormone therapy	67 women were diagnosed between 2005 and 2011, 3 were diagnosed between 1981 and 1999	Center of Epidemiologic Studies Depression Scale (CES-D)	FACT-G EORTC QLQ-BR23	Depression was significantly correlated with QoL	Body image, physical attractiveness, and femininity, sociodemographic and clinical variables
Cheng A.S.K., 2016 [43]	Cross-sectional	China	90 women aged between 18 and 60 years. 30 were breast cancer survivors, 30 had musculoskeletal conditions, and 30 healthy women. 86.7% = < or completed secondary education; 13.3% = > secondary education 53.3% were married	42.3% = Early Stage 30.8% = Mid Stage 26.9% = Late Stage 10% = Surgery 13.3% = Radiation 10% = Surgery + Radiation 66.7% = Surgery + Radiation + Chemotherapy	Time since completing treatment was 36 months (SD = 33)	Hospital Anxiety and Depression Scale (HADS)	EORTC QLQ-C30	There was no significant differences in depression among the groups	Anxiety, cognitive symptoms, work limitations and sociodemographic and clinical variables
DeShields, T., 2006 [24]	Longitudinal	United States	84 women aged 28–87 (mean = 56, SD = 14). 73% = Caucasian,27% = African American 38% = < or completed secondary education; 62% = > secondary education 61% were married	10% = Stage 0 44% = Stage 1 39% = Stage 2 7% = Stage 3 77% = Lumpectomy 23% = Mastectomy; 48% = Chemotherapy 70% = Hormonal therapy	1 week prior to radiation treatment. 3 and 6 months post treatment	Center of Epidemiologic Studies Depression Scale (CES-D)	FACT-B	At time 1 the Depressed, groups demonstrated significantly worse QoL than the Never Depressed group. Findings were similar at Time 2. At Time 3, the Recover group demonstrated equivalent QoL to the Never Depressed group, while the other groups exhibited significantly worse QoL	Sociodemographic and clinical variables
Goyal N. G, 2018 [23]	Longitudinal	United States	565 women aged 25–96 (mean = 55, SD = 16). 90% Caucasian; 10% = Non-Caucasian. 63% were college educated 72% were partnered	52% = Stage 1 40% = Stage 2 8% = Stage 3 36% = Mastectomy 67% = Chemotherapy 72% = Radiation 73% = Hormonal therapy	Baseline data was collected within 8 months of diagnosis. Follow-up data was collected at 6, 12, and 18 months post baseline	Becks Depression Inventory	FACT-B	Those in the “consistently high” QoL trajectory had lower depression compared to all other groups	Social support, coping, spirituality, optimism, Illness Intrusiveness Rating Scale and sociodemographic and clinical variables
Simone, S.M. H, 2013 [31]	Cross-sectional	China	148 Chinese women aged on average 50.5 (SD = 9.1). 7.4% = no formal education, 27.1% = primary education, 51.3% = secondary education, 14.1% = post-secondary. 76.2% were married/co-habiting	6.7% = Stage 0 12.3% = Stage 1 43.5% = Stage 2 27.9% = Stage 3 9.7% = Stage 4 92.6% = Surgery 84.0% = Chemotherapy 78.4% = Radiotherapy 47.2% = Hormonal therapy 0.4% = Traditional Chinese medicine	Recruitment occurred from 2010 to 2011. Treatment had been completed within that last year	Hospital Anxiety and Depression Scale (HADS) Cantonese/Chinese version	FACT-G	Depression was a significant predictor of physical wellbeing functional wellbeing and social/family wellbeing	Anxiety, sociodemographic and clinical variables
Janz N.K., 2014 [26]	Longitudinal	United States	772 women aged on average 59.1 (SD = 13). 47.3% = Caucasian; 16.8% = Black; 36.9% = Latina. 21% = < secondary education; 20.9% = completed secondary education; 58.1% = > secondary education 55% were married	55.3% = Stage 1 36.0% = Stage 2 8.7% = Stage 3 40.7% = Mastectomy 57.8% = Lumpectomy 64.5% = Radiation 45.2% = Chemotherapy	Data was taken 9 months post diagnosis. Follow-up occurred 4 years post diagnosis	Depression history	FACT-B- Emotional well-being (EWB) subscale	Compared with women without a history of depression, women with a history of depression and women with current depression were significantly more likely to report EWB declines	Social support, spirituality, appraisal and sociodemographic and clinical variables
Kim, S.H.., 2008 [44]	Cross-sectional	Korea	1933 women aged on average 44 (SD = 9.3). 62.4% = < 50; 37.6% = > 50; 29.3% = < secondary education; 41.3% = completed secondary education; 29.4% = > secondary education 84.9% were married	7.9% = Stage 0; 34.3% = Stage 1; 48.8% = Stage 2; 8.9% = Stage 3 32.9% = Surgery 67.0% = Mastectomy 28.6% = Chemotherapy 13.1% = Hormone therapy 34.8% = Chemotherapy + Hormone therapy	The average time since surgery was 6 years (SD = 4)	Becks Depression Inventory	EORTC QLQ-C30 EORTC QLQ-BR23	There were large differences in mean scores (lower scores) for those with depression vs. not for global QOL, emotional functioning, and future perspective scales and smaller mean differences in sexual functioning or sexual enjoyment scores	Fatigue, sociodemographic and clinical variables
***Anxiety***									
Akechi, T., 2015 [45]	Cross-sectional	Japan	146 women aged 27–87 years (mean = 57, SD 11) 38% > 12 years education. 75% were married	9% = Stage 0 49% = Stage 1 38% = Stage 2 4% = Stage 3 99% = Surgery; 35% = Chemotherapy 1% = Trastuzumab 71% = Hormonal therapy 43% = Radiation therapy	February 2006–February 2007. 733–4131 days since diagnosis (mean = 1569, SD = 786)	Hospital Anxiety and Depression Scale (HADS)	EORTC QLQ-C 30	Anxiety level was significantly correlated with all QoL measures -global health status, physical, role emotional cognitive and social functioning	Depression, perceived needs and sociodemographic and clinical variables
Cheng A.S.K., 2016 [43]	Cross-sectional	China	90 women aged between 18 and 60 years. 30 were breast cancer survivors, 30 had musculoskeletal conditions, and 30 healthy women. 86.7% = < or completed secondary education; 13.3% = > secondary education. 53.3% were married	42.3% = Early Stage 30.8% = Mid Stage 26.9% = Late Stage 10% = Surgery 13.3% = Radiation 10% = Surgery + Radiation 66.7% = Surgery + Radiation + Chemotherapy	Time since completing treatment was 36 months (SD = 33)	Hospital Anxiety and Depression Scale (HADS)	EORTC QLQ-C30	There was no significant differences in anxiety among the groups	Depressiom, cognitive symptoms, work limitations and sociodemographic and clinical variables
Simone, S.M. H, 2013 [31]	Cross-sectional	China	148 Chinese women aged on average 50.5 (SD = 9.1). 7.4% = no formal education, 27.1% = primary education, 51.3% = secondary education, 14.1% = post-secondary. 76.2% were married/co-habiting	6.7% = Stage 0 12.3% = Stage 1 43.5% = Stage 2 27.9% = Stage 3 9.7% = Stage 4 92.6% = Surgery 84.0% = Chemotherapy 78.4% = Radiotherapy 47.2% = Hormonal therapy 0.4% = Traditional Chinese medicine	Recruitment occurred from 2010 to 2011. Treatment had been completed within that last year	Hospital Anxiety and Depression Scale (HADS)- Cantonese/Chinese version	FACT-G	Anxiety was a significant predictor of physical, functional and emotional wellbeing	Depression and sociodemographic and clinical variables
***Positive and Negative Affect***									
Kessler, T. A. 2002 [46]	Cross-sectional	United States	148 women aged on average 52.4 years (SD = 11.56). 85% = Caucasian; 11% = African American; 3% = Hispanic; 1% = Other. 5% = < secondary education; 38% = completed secondary education; 57% = > secondary education 72% were married	24% = Mastectomy 18% = Mastectomy + Chemotherapy 11% = Mastectomy + Hormone therapy 1% = Mastectomy + Radiation 7% = Lumpectomy + Radiation	Time since diagnosis was between 0.3–19 years (M = 54, SD = 6)	Positive and Negative Affect Scale (PANAS)	QOLM	Positive affect was related positively to QoL and negative affect was related negatively to QoL	
***Coping***									
Dura-Ferrandis E, 2016 [28]	Longitudinal	United States	1280 women aged 65–91 (mean = 77, SD = 9). 88.1% = Caucasian; 11.9% = Non-Caucasian 42.1% = < or completed secondary education; 57.9% = > secondary education 55.3% were married	45.6% = Stage 1 31.2% = Stage 2a 23.2% = Stage 2b or higher 67.6% = Breast Cancer Surgery 32.4% = Mastectomy 57.0% = Hormonal therapy only 43.0% = Chemotherapy	Recruitment was conducted from January, 2004 and April 2011 with follow-up in June, 2011. Baseline data was collected nearly 2 months after last surgery. Follow-up data was collected 6 and 12 months after the baseline interview and annually for up to 7 years	Brief COPE	EORTC QLQ-C30	The accelerated emotional decline group (vs maintained high) were more likely to use disengagement coping strategies and self-distraction	Social support. Optimism and sociodemographic variables
Goyal N. G, 2018 [23]	Longitudinal	United States	565 women aged 25–96 (mean = 55, SD = 16). 90% Caucasian; 10% = Non-Caucasian. 63% were college educated 72% were partnered	52% = Stage 1 40% = Stage 2 8% = Stage 3 36% = Mastectomy 67% = Chemotherapy 72% = Radiation 73% = Hormonal therapy	Baseline data was collected within 8 months of diagnosis. Follow-up data was collected at 6, 12, and 18 months post baseline	Brief COPE	FACT-B	Those in the “consistently high” QoL trajectory had lower passive coping scores compared to all other groups	Social support, depression, spirituality, optimism, Illness Intrusiveness Rating Scale and sociodemographic and clinical variables
Paek M, 2016 [47]	Cross-sectional	United States	156 women aged on average 55.29 years (SD = 9.69). 55% = Chinese, 45% = Korean 26.9% = < or completed secondary education; 73.1% = > secondary education 75% were married	7.1% = Stage 0 35.9% = Stage 1 43.6% = Stage 2 13.5% = Stage 3 68% = Chemotherapy 53% = Mastectomy	There was an average of 3.49 years since diagnosis (SD = 1.47)	Family Crisis Oriented Personal Scale (F-COPES)	FACT-B- Emotional Well-Being subscale SF-36- Vitality subscale	Use of external family coping had a direct positive effect on mental health outcomes, whereas internal family coping had no effect	Negative self-image and life stress, family communication strain
Paek, M., 2016 [25]	Longitudinal	United States	637 women aged 26–97 years (mean = 55, SD = 16). 89.6% = Caucasian; 5.5% = Black; 4.9% = Other 12.6% = < or completed secondary education; 87.4% = > secondary education 71.9% were married	52.4% = Stage 1 39.7% = Stage 2 7.8% = Stage 3 72.2% = Radiotherapy 66.4% = Chemotherapy 64.2% = Lumpectomy 35.8% = Mastectomy	Recruitment occurred from 2002 to 2006. Baseline (Time 1) data was taken 1 and 3 months since diagnosis (mean = 5, SD = 3). Time 2 was 12–20 months post diagnosis. Time 3 was 18–26 months post diagnosis	Brief COPE	FACT-B	The direct paths from Time 1 negative coping to Time 2 QoL and Time 2 QoL to Time 3 negative coping were both statistically significant. No reciprocal relation between QoL and positive coping	Sociodemographic and clinical variables
Avis N.E. (2005) [41]	Cross-sectional	United States	202 women between the age of 25 and 50 years (mean 43.5 years). 96% were White. 20.3% = < or completed secondary education; 79.7% = > secondary education 81% were married/partner	43.4% = Mastectomy 75.1% = Chemotherapy 69.6% = Radiation therapy	Diagnosed with their first breast cancer in the previous 3 years and were at least 4 months after diagnosis	Ways of Coping	FACT-B Ladder of Life (overall QoL)	Keeping to self was negatively associated with functional well-being. Positive cognitive restructuring, making change and being prepared was positively associated with QoL. Wishful thinking was negatively associated with QoL	Social support, sociodemographic and clinical variables
***Confidence and self-efficacy***									
Carver C.S. et al. 2006 [13]	Longitudinal	United States	163 women with a mean age of 51.4 (SD = 10.61). 70% = Caucasian, 20% = Hispanic; 10% = African American. 72% were married	3% = Stage 0 62% = Stage 1 35% = Stage 2 53% = Lumpectomy 47% = Mastectomy 31% = Chemotherapy 50% = Radiation 56% = Tamoxifen 25% = Reconstruction	Recruitment between 1988 and 1995 and 1994–1996. Data collection in 2001. 5–13 years since surgery	Question- confidence about remaining cancer free	QLACS	In multivariate analysis confidence was significantly associated with cognitive impairment (subscale of QLACS)	Optimism, social support and sociodemographic, clinical and treatment variables
DiSipio T et al., 2009 [35]	Cross-sectional	Australia	323 women. 67% of women were aged ≥50 years. 202 regional based and 121 rural. 54% = < secondary education; 32% = completed secondary education; 14% = > secondary education 77% were married	61% = Complete local excision 39% = Mastectomy / partial / radical Adjuvant treatment 18% = No treatment 82% = Chemotherapy / Radiotherapy	Recruitment between April 2006 March 2012 post diagnosis	Health efficacy- Self-rated Abilities for Health Practices	FACT-B	Poorer health self-efficacy was associated with a lower QoL	Social support, amount of stress, perceived handling of stress and sociodemographic and clinical variables
Pedro L.W. (2001) [39]	Cross-sectional	United States	62 women aged ≥60 years. Majority were married, retired, white and college-educated.	Majority surgery or a combination of surgery and radiation	5 to 10 years beyond initial diagnosis and disease and recurrence free	Rosenberg Self-Esteem Scale and Rosenbaum Self-Control Schedule	QLI-CV	A statistically significant positive correlation was found between self-esteem and QoL. In multiple regression Learned resourcefulness was found to be related inversely to QoL	Social support
***Spirituality***									
Goyal N. G, 2018 [23]	Longitudinal	United States	565 women aged 25–96 (mean = 55, SD = 16). 90% Caucasian; 10% = Non-Caucasian. 63% were college educated 72% were partnered	52% = Stage 1 40% = Stage 2 8% = Stage 3 36% = Mastectomy 67% = Chemotherapy 72% = Radiation 73% = Hormonal therapy	Baseline data was collected within 8 months of diagnosis. Follow-up data was collected at 6, 12, and 18 months post baseline	Functional Assessment of Chronic Illness Therapy–Spiritual Well-Being scale	FACT-B	Those in the “consistently high” QoL trajectory had higher scores on meaning/peace and role of faiths compared to all other groups	Social support, depression, coping, optimism, Illness Intrusiveness Rating Scale and sociodemographic and clinical variables
Janz N.K., 2014 [26]	Longitudinal	United States	772 women aged on average 59.1 (SD = 13). 47.3% = Caucasian; 16.8% = Black; 36.9% = Latina. 21% = < secondary education; 20.9% = completed secondary education; 58.1% = > secondary education 55% were married	55.3% = Stage 1 36.0% = Stage 2 8.7% = Stage 3 40.7% = Mastectomy 57.8% = Lumpectomy 64.5% = Radiation 45.2% = Chemotherapy	Data was taken 9 months post diagnosis. Follow-up occurred 4 years post diagnosis	System of Beliefs Inventory (SBI-15R)	FACT-B- Emotional well-being subscale	A higher mean score on the beliefs and practices subscale of Spiritual Beliefs Inventory–15R was associated with emotional decline	Social support, depression, appraisal and sociodemographic and clinical variables
Manning-Walsh J, 2005 [48]	Cross-sectional	United States	100 women aged 30–74 years (mean = 45.98, SD = 8.85) 93% = Caucasian; 3% = American Indian; 2% = Hispanic; 2% = Other 2% = < secondary education; 23% = completed secondary education; 73% = > secondary education 74% were married	48% = Stage 1 41% = Stage 2 5% = Stage 3 4% = Stage 4 2% = Missing 51% = Mastectomy 48% = Lumpectomy 70% = Chemotherapy 68% = Radiation therapy 41% = Hormone therapy	Recruitment occurred in 2000. 1–24 months post-surgery	Religious Coping (RCOPE) - negative coping subscale	FACT-B	A negative significant correlation between spiritual struggle and QoL	
Wildes, K.A. 2009 [49]	Cross-sectional	United States	117 Latina women aged on average 57.2 years (SD = 10.21). 64.1% = Mexican/ American/Chicano; 1.7% = Central American; 0.9% = Puerto Rican; 0.9% = Cuban; 2.6% = South American; 26.5% = Other Latino/Hispanic; 3.4% = Other. 35% = < secondary education; 35% = completed secondary education; 30% = > secondary education.50.4% were married	99.1% = Surgery	92% were diagnosed less than 10 years ago	Systems of Belief Inventory (SBI-15R)	FACT–B	Spirituality was significantly associated with functional wellbeing	Sociodemographic and clinical variables
***Optimism***									
Carver C.S. et al. 2006 [13]	Longitudinal	United States	163 women with a mean age of 51.4 (SD = 10.61). 70% = Caucasian, 20% = Hispanic; 10% = African American. 72% were married	3% = Stage 0 62% = Stage 1 35% = Stage 2 53% = Lumpectomy 47% = Mastectomy 31% = Chemotherapy 50% = Radiation 56% = Tamoxifen 25% = Reconstruction	Recruitment between 1988 and 1995 and 1994–1996. Data collection in 2001. 5–13 years since surgery	Life Orientation Test	QLACS	In multivariate analysis optimism was significantly inversely associated with negative feelings, lack of positive feelings, sexual impairment social avoidance, fatigue, lack of benefits, recurrence distress and appearance worries	Social Support, cancer confidence and sociodemographic, clinical and treatment variables
Dura-Ferrandis E, 2016 [28]	Longitudinal	United States	1280 women aged 65–91 (mean = 77, SD = 9). 88.1% = Caucasian; 11.9% = Non-Caucasian 42.1% = < or completed secondary education; 57.9% = > secondary education. 55.3% were married	45.6% = Stage 1 31.2% = Stage 2a 23.2% = Stage 2b or higher 67.6% = Breast Cancer Surgery 32.4% = Mastectomy 57.0% = Hormonal therapy only 43.0% = Chemotherapy	Recruitment was conducted from January, 2004 and April 2011 with follow-up in June, 2011. Baseline data was collected nearly 2 months after last surgery. Follow-up data was collected 6 and 12 months after the baseline interview and annually for up to 7 years.	Life Orientation Test	EORTC QLQ-C30	Those in the accelerated emotional decline group (vs maintained high) were significantly less optimistic	Social support, coping and sociodemographic variables
Goyal N. G, 2018 [23]	Longitudinal	United States	565 women aged 25–96 (mean = 55, SD = 16). 90% Caucasian; 10% = Non-Caucasian. 63% were college educated 72% were partnered	52% = Stage 1 40% = Stage 2 8% = Stage 3 36% = Mastectomy 67% = Chemotherapy 72% = Radiation 73% = Hormonal therapy	Baseline data was collected within 8 months of diagnosis. Follow-up data was collected at 6, 12, and 18 months post baseline	Life Orientation Test	FACT-B	Those in the “consistently high” QoL trajectory had higher scores on optimism compared to all other groups (*p* < 0.001)	Social support, depression, coping, spirituality, Illness Intrusiveness Rating Scale and sociodemographic and clinical variables
Northouse, L.L (1999) [38]	Cross-sectional	United States	98 African American women aged 29–81 years (mean = 55, SD = 18). Average education was 13 years (SD = 6). 41% were married	70% = Mastectomy	The average time since diagnosis was 6 years (SD = 3). Time since diagnosis ranged from 1 to 15 years	Life Orientation Test	FACT-B	Optimism was not significantly associated with QoL	Symptom distress, current concerns, family functioning, appraisal of illness and sociodemographic and clinical variables
Petersen L.R. (2008) [50]	Cross-sectional	United States	268 women aged 32–95 years (mean = 71, SD = 11.90)	10.9% = Stage 0 63.3% = Stage 1 21.3% = Stage 2 4.5% = Stage 3	QoL was measured on average 8 years after diagnosis. The mean number of years between completion of MMPI to breast cancer diagnosis was 10 (SD = 8.4) and 18.3 (SD = 9.2) between MMPI and SF-36 completion	The Minnesota Multiphasic Personality Inventory (MMPI) – optimism-pessimism (PSM) scale	SF-36	Women with a pessimistic explanatory style had significantly lower mental QoL compared to those with a non-pessimistic style	
***Future perspective and appraisal***									
Sammarco, A., 2008 [32]	Cross-sectional	United States	89 Latina breast cancer survivors with a mean age of 57.35 years (SD = 12.74, range 30–86 years). 65% = Caucasian; 35% = Latina.7% = < secondary education; 41% = completed secondary education; 52% = > secondary education. 61% were married	17% = Surgery only 6% = Adjuvant only 77% = Both	Breast cancer treatment was completed between 1 and 35 years prior (mean = 4.99 years, SD = 4.73)	Mishel Uncertainty in Illness Scale-Community (MUIS-C)	QLI-CV	Decreased uncertainty was associated with improved QoL	Social support
Northouse, L.L (1999) [38]	Cross-sectional	United States	98 African American women aged 29–81 years (mean = 55, SD = 18). Average education was 13 years (SD = 6). 41% were married	70% = Mastectomy	The average time since diagnosis was 6 years (SD = 3). Time since diagnosis ranged from 1 to 15 years	Appraisal of Illness Questionnaire	FACT-B	Appraisal of illness mediated the influence of optimism and current concerns and partially mediated the influence of symptom distress on women’s QoL	Optimism, symptom distress, current concerns, family functioning, and sociodemographic and clinical variables
Farren, A. T, 2010 [51]	Cross-sectional	United States	104 women aged 28–81 years (mean = 53). 92% were Caucasian. 40% = < or completed secondary education; 60% = > secondary education. 69% were married		The majority of women completed treatment (52%) more than 5 years prior to the study	Power as Knowing Participation in Change Tool (PKPCT) Mishel Uncertainty in Illness Scale – Community Form (MUISC) Self-Transcendence Scale (STS)	QLI-CV	39% of the variance in QoL could be explained by power, uncertainty and self-transcendence when considered together Uncertainty and self-transcendence made a statistically significant contribution to the explained variance, power did not	Sociodemographic variables
Janz N.K., 2014 [26]	Longitudinal	United Stat es	772 women aged on average 59.1 (SD = 13). 47.3% = Caucasian; 16.8% = Black; 36.9% = Latina. 21% = < secondary education; 20.9% = completed secondary education; 58.1% = > secondary education 55% were married	55.3% = Stage 1 36.0% = Stage 2 8.7% = Stage 3 40.7% = Mastectomy 57.8% = Lumpectomy 64.5% = Radiation 45.2% = Chemotherapy	Data was taken 9 months post diagnosis. Follow-up occurred 4 years post diagnosis.	Recurrence information, likelihood and worry about recurrence, decision regret	FACT-B- Emotional well-being subscale	Women who did not receive enough information about the risk of breast cancer recurrence, perceived that their likelihood of breast cancer recurrence was quite/very likely and had higher worry about recurrence (Time 1 to Time 2) were significantly more likely to report emotional decline. No significant differences were observed for decision regret	Social support, depression, spirituality and sociodemographic and clinical variables
Koch, L 2014 [29]	Cross-sectional	Germany	2671 women aged on average 65 (SD = 9.7). 52% = < secondary education 48% = completed or > secondary education 66% were married	45% = Stage 1 47% = Stage 2 8% = Stage 3 < 1% = Stage 4 32% = Mastectomy 68% = Breast Cancer Surgery 60% = Chemotherapy 84% = Radiation 49% = Hormonal therapy	Recruitment took place from 2009 to 2010. The mean time since diagnosis was 2 years (ranged 5–16)	Fear of Progression Questionnaire-Short Form	EORTC QLQ-C30- EORTC QLQ-BR23	Fear of progression was significantly associated with global, physical, functional, social, emotional and cognitive QoL	Sociodemographic and clinical variables
Taylor, T.R., 2012 [30]	Cross-sectional	United States	51 women aged 31–87 (mean = 64, SD = 13). 100% were African American. 13.7% = completed secondary education; 84.3% = > secondary education; 2% = Missing. 35.3% were married	27.5% = Stage 0 43.1% = Stage 1 19.6% = Stage 2 9.8% = Stage 3 45.1% = Breast Cancer Surgery 52.9% = Mastectomy 33.3% = Surgery + Radiation 15.7% = Surgery + Chemotherapy 31.4% = Surgery + Chemotherapy + Radiation 7.8% = Surgery + Hormone + Other	Time since diagnosis ranged from 1 to 18 years (mean = 2, SD = 3)	Concerns of Recurrence Scale	FACT-B	Overall fear of recurrence was negatively related to QoL	
Ganz P.A. (2003) [52]	Cross-sectional	United States	577 women aged 30–61.6 years (mean = 49.5). 70.2% = Caucasian; 11.6% = African American; 7.3% = Hispanic; 8.5% = Asian; 2.4% = Other 6.3% = < or completed secondary education; 93.7% = > secondary education. 70.3% were married	55.8% = Lumpectomy 44.2% = Mastectomy 62% = Adjuvant chemotherapy 37.4% = Tamoxifen	On average 6 years after breast cancer diagnosis	Vulnerability	SF-36 general health perceptions scale Ladder of Life	Feeling vulnerable was significantly associated with poorer health perceptions and QoL	Physical and emotional functioning, sociodemographic and clinical variables
***Impact of Events***									
Lewis J., 2001 [37]	Cross-sectional	United States	64 women aged between 30 and 81 years (mean = 59.2 SD = 9.8). 80% = Caucasian; 20% = African American / Hispanic / Asian / West Indian 6.5% = < secondary education 28% = completed secondary education; 65.5% = > secondary education 66% were married	89% = Chemotherapy / Radiation 71% = Mastectomy 23% = Lumpectomy	Last treatment ranged from 1 to 15 years prior (mean = 7)	Impact of Events Survey (IES)- Intrusion subscale.	SF-36	A higher frequency of intrusive thoughts was associated with poorer physical QoL and mental QoL	Social support and sociodemographic variables
Goyal N. G, 2018 [23]	Longitudinal	United States	565 women aged 25–96 (mean = 55, SD = 16). 90% Caucasian; 10% = Non-Caucasian. 63% were college educated 72% were partnered	52% = Stage 1 40% = Stage 2 8% = Stage 3 36% = Mastectomy 67% = Chemotherapy 72% = Radiation 73% = Hormonal therapy	Baseline data was collected within 8 months of diagnosis. Follow-up data was collected at 6, 12, and 18 months post baseline	Illness Intrusiveness Rating Scale	FACT-B	Those in the “consistently high” QoL trajectory had lower scores on illness intrusiveness compared to all other groups	Social support, depression, coping, spirituality, optimism and sociodemographic and clinical variables
Bouskill K. (2016) [53]	Cross-sectional	Austria	152 women aged on average 48.78 (SD 8.79). 12% = < secondary education; 46% = completed secondary education; 40% = > secondary education; 2% = Missing. 67% were married/long-term partner		On average women diagnosed 13 years prior to study start date (2001) (SD 3.17)	Impact of Cancer scale	SF-36- the physical and the mental component summary	The positive impact of cancer was associated with an increase in physical QoL while the negative impact of cancer was associated with a decrease in physical QoL and mental QoL	Sociodemographic and clinical variables
***Stress***									
Morrill, E.F., 2008 [54]	Cross-sectional	United States	161 women aged 36–87 years (mean = 59, SD = 10.6). 85% = Caucasian; 12% = African American.3% = < secondary education44% = completed secondary education; 53% = > secondary education. 73% were married	55% = Stage 1 43% = Stage 2 99% = Surgery 54% = Chemotherapy 62% = Radiation 67% = Tamoxifen	The average time since diagnosis was 4 years (SD = 1)	Post-traumatic Stress Disorder Checklist Civilian Version (PCL-C)	FACT-B	Post-traumatic stress symptoms were significantly associated with QoL and depression	Posttraumatic growth, sociodemographic and clinical variables
Northouse, L.L (1999) [38]	Cross-sectional	United States	98 African American women aged 29–81 years (mean = 55, SD = 18). Average education was 13 years (SD = 6). 41% were married	70% = Mastectomy	The average time since diagnosis was 6 years (SD = 3). Time since diagnosis ranged from 1 to 15 years	Omega Screening Questionnaire (OSQ)	FACT-B	Symptom distress made a significant independent contribution to women’s QoL. The influence of current concerns on women’s QoL was mediated by appraisal of illness	Optimism, appraisal of illness, family functioning, and sociodemographic and clinical variables
Ashing –Giwa K.T.. 2010 [33]	Cross-sectional	United States	703 participants aged 29–91 years (mean = 55, SD = 13). European- (*n* = 179), African- (*n* = 135), Latina- (*n* = 183), and Asian- (*n* = 206) Americans. 14.4% = < secondary education; 10.8% = completed secondary education; 74.8% = > secondary education	11.1% = Stage 0 36.7% = Stage 1 38.5% = Stage 2 13.7% = Stage 3 58.5% = Lumpectomy/other; 38.4% = Mastectomy; 15.6% = Mastectomy and reconstruction; 57.8% = Chemotherapy; 66.0% = Radiation	1–5 years since diagnosis (mean = 3 years)	Life burden- Life Stress Scale	FACT-B- Physical and emotional well-being scale SF-36 -general health perception sub-domain and pain sub-domain	Life burden was significantly related to physical and psychological QoL	Social support. Health care system- patient-doctor relationship, comfort in health care system and diagnostic care delay and sociodemographic variables
DiSipio T et al., 2009 [35]	Cross-sectional	Australia	323 women. 67% of women were aged ≥50 years. 202 regional based and 121 rural. 54% = < secondary education; 32% = completed secondary education; 14% = > secondary education. 77% were married	61% = Complete local excision 39% = Mastectomy / partial / radical Adjuvant treatment 18% = No treatment 82% = Chemotherapy / Radiotherapy	Recruitment between April 2006 March 2012 post diagnosis	Amount of stress and perceived handling of stress	FACT-B + 4 (plus arm morbidity)	Amount of stress and perceived handling of stress was associated with a lower QoL	Social support, health care service needs, overall health self-efficacy and sociodemographic and clinical variables
Paek M, 2016 [47]	Cross-sectional	United States	156 women aged on average 55.29 years (SD = 9.69). 55% = Chinese, 45% = Korean 26.9% = < or completed secondary education; 73.1% = > secondary education. 75% were married	7.1% = Stage 0 35.9% = Stage 1 43.6% = Stage 2 13.5% = Stage 3 68% = Chemotherapy 53% = Mastectomy	There was an average of 3.49 years since diagnosis (SD = 1.47).	Negative Self-Image (FACT-B) Urban Life Stress Scale. Secondary Stressor The Family Communication Scale of the Family Adaptability and Cohesion Evaluation (FACES-IV) and the Family Avoidance of Communication about Cancer (FACC)	FACT-B- Emotional Well-Being subscale SF-36- Vitality subscale	Greater primary stressors (negative self-image and life stress) were associated with poorer mental health Greater secondary stress (family communication strain) had negative effects on mental health outcomes	Coping
***Post-traumatic Growth***									
Morrill, E.F., 2008 [54]	Cross-sectional	United States	161 women aged 36–87 years (mean = 59, SD = 10.6). 85% = Caucasian; 12% = African American.3% = < secondary education44% = completed secondary education; 53% = > secondary education. 73% were married	55% = Stage 1 43% = Stage 2 99% = Surgery 54% = Chemotherapy 62% = Radiation 67% = Tamoxifen	The average time since diagnosis was 4 years (SD = 1).	Posttraumatic Growth Inventory	FACT-B	Posttraumatic growth was significantly associated with QoL Posttraumatic growth was not related to depressive symptoms but interacted with post-traumatic stress symptoms in predicting depressive symptoms	Post-traumatic stress symptoms, sociodemographic and clinical variables
Bellizzi K.M. (2009) [27]	Longitudinal	United States	802 women aged on average 57.2 years (SD = 10.1). 62.3% = Caucasian; 12.2% = Hispanic; 25.5% = Black. 25.4% = < or completed secondary education;74.6% = > secondary education 56% were married	32.4% = Surgery only 36.8% = Surgery + Radiation 9.1% = Surgery + Chemotherapyh 21.7% = Surgery + Radiation + Chemotherapy	Baseline questionnaire – on average 6.1 months following diagnosis, Time 2–24 months after baseline, Time 3–35 months after baseline	Posttraumatic Growth Inventory	SF-36 – physical component score (PCS) and mental component score (MCS)	Posttraumatic growth was significantly associated with a lower mental QoL No association with physical QoL	Religiosity and sociodemographic and clinical variables
***Cognitive Symptoms***									
Cheng A.S.K., 2016 [43]	Cross-sectional	China	90 women aged between 18 and 60 years. 30 were breast cancer survivors, 30 had musculoskeletal conditions, and 30 healthy women. 86.7% = < or completed secondary education; 13.3% = > secondary education. 53.3% were married	42.3% = Early Stage 30.8% = Mid Stage 26.9% = Late Stage 10% = Surgery 13.3% = Radiation 10% = Surgery + Radiation 66.7% = Surgery + Radiation + Chemotherapy	Time since completing treatment was 36 months (SD = 33)	Cognitive Symptom Checklist-Work	EORTC QLQ-C30	The number of cognitive symptoms was significantly higher in breast cancer survivors, especially for the symptoms related with working memory. The cognitive limitations were significantly associated with QoL	Anxiety, depression, work limitations and sociodemographic and clinical variables
***Work Limitations***									
Cheng A.S.K., 2016 [43]	Cross-sectional	China	90 women aged between 18 and 60 years. 30 were breast cancer survivors, 30 had musculoskeletal conditions, and 30 healthy women. 86.7% = < or completed secondary education; 13.3% = > secondary education. 53.3% were married	42.3% = Early Stage 30.8% = Mid Stage 26.9% = Late Stage 10% = Surgery 13.3% = Radiation 10% = Surgery + Radiation 66.7% = Surgery + Radiation + Chemotherapy	Time since completing treatment was 36 months (SD = 33)	Work Limitation Questionnaire (WLQ)	EORTC QLQ-C30	There was no significant difference in the overall WLQ productivity loss score among the three groups	Depression, anxiety, cognitive symptoms, and sociodemographic and clinical variables
***Health care system***									
Ashing –Giwa K.T.. 2010 [33]	Cross-sectional	United States	703 participants aged 29–91 years (mean = 55, SD = 13). European- (n = 179), African- (n = 135), Latina- (n = 183), and Asian- (n = 206) Americans. 14.4% = < secondary education; 10.8% = completed secondary education; 74.8% = > secondary education	11.1% = Stage 0 36.7% = Stage 1 38.5% = Stage 2 13.7% = Stage 3 58.5% = Lumpectomy/other; 38.4% = Mastectomy; 15.6% = Mastectomy and reconstruction; 57.8% = Chemotherapy; 66.0% = Radiation	1–5 years since diagnosis (mean = 3 years)	Health care system- patient-doctor relationship, comfort in health care system and diagnostic care delay	FACT-B- Physical and emotional well-being scale SF-36 -general health perception sub-domain and pain sub-domain	Only European and Latina-Americans showed positive relationships between patient-doctor relationship and psychological well-being. European-Americans showed the direct impact of diagnostic care delay on physical QoL	Social support, life burden and sociodemographic variables

Functional Assessment of Cancer - Breast Cancer (FACT-B), Medical Outcomes Study Short Form (SF-36), The European Organisation of Research and Treatment of Cancer Quality of Life Questionnaire (EORTC QLQ-C30), The European Organisation of Research and Cancer Treatment Quality of Life Questionnaire - Breast Cancer (QLQ-BR23), Functional Assessment of Cancer Therapy - General (FACT-G), Quality of Life Index - Cancer Version (QLI-CV), Quality of Life Measurement (QoL-M), Quality of Life in Adult Cancer Survivors (QLACS), Quality of Life Cancer Survivor Version (QOL-CS), Global Life Satisfaction Scale (GLSS)

### QoL measures

There were 12 different validated QoL outcome measures utilised by the various studies in the scoping review (Table 3). The Functional Assessment of Cancer - Breast Cancer (FACT-B) was the most frequently used QoL measure (13 studies) [23–26, 30, 33, 35, 38, 41, 47–49, 54]. Two studies applied the Functional Assessment of Cancer Therapy – General (FACT-G), without the Breast Cancer Subscale (BC Subscale) [31, 42]. The SF-36 was used in 8 studies [27, 33, 36, 37, 47, 50, 52, 53]. The EORTC QLQ-C30 was used to assess QoL in 6 studies [28, 29, 43–45, 56] and EORTC QLQ-BR23 in 3 studies [29, 42, 44]; with 2 of these studies using both the EORTC QLQ-C30 and QLQ-BR23 [29, 44]. Three studies applied the Quality of Life Index Cancer Version (QLI-CV) [32, 39, 51]. The remaining QoL measures were used less frequently (≤ 2 studies). Seven studies (21%) used more than one measure of QoL as an outcome [29, 33, 41, 42, 44, 47, 52].

**Table 3:** Frequency of QoL measures

QoL measure	Frequency used	Primary Author, Year
Functional Assessment of Cancer - Breast Cancer (FACT-B). This 44-item self-report instrument was designed to measure multidimensional QoL in patients with breast cancer. The FACT-B includes the FACT-G with four domains covering physical, emotional, social/family and functional well-being and a Breast Cancer Subscale measuring the adverse effects of endocrine therapy [21]	13	Ashing-Giwa, K. T, 2010 [33], DiSipio, T., 2009 [35], Goyal, N., 2018 [23], Janz, N., 2014 [26], Manning-Walsh, J., 2005 [48], Morrill, F., 2008 [54], Northouse, L., 1999 [38], Paek, M.S., 2016 [47], Paek, M.S., 2016 [25], Taylor, T., 2012 [30] Avis N.E. 2005 [41]. DeShields T 2006 [24], Wildes, K., 2009 [49]
Medical Outcomes Study Short Form (SF-36). This questionnaire consists of an eight-item scale. The scales consist of: physical functioning, general health, vitality, social functioning, emotional, and mental health. All scales load onto one of two distinct concepts, the physical component summary or the mental component summary [55]	8	Lewis, J., 2001 [37], Paek, M.S., 2016 [47], Huang, C.Y., 2013 [36], Ashing-Giwa, K. T, 2010 [33], Ganz P.A., 2003 [52], Petersen, L.R.,2008 [50], Bouskill, K., 2016 [53], Bellizzi K.M., 2010 [27]
The European Organisation of Research and Treatment of Cancer Quality of Life Questionnaire (EORTC QLQ-C30). This questionnaire consists of 30 items incorporating nine multi-item scales: five functional scales (physical, role, cognitive, emotional, and social); three symptom scales (fatigue, pain, and nausea and vomiting); and a global health and quality-of-life scale [22]	6	Akechi, T., 2015 [45], Cheng, A.S.K., 2016 [43], Dura-Ferrandis, E., 2016 [28], Kim, S. H.., 2008 [44], Edib Z, 2016 [56], Koch, L, 2014 [29]
The European Organisation of Research and Cancer Treatment Quality of Life Questionnaire - Breast Cancer (QLQ-BR23). This questionnaire consists of 23-items covering symptoms and side effects related to different treatment modalities, body image, sexuality, and future perspective [57]	3	Koch, L, 2014 [29], Begovic-Juhant, A., 2012 [42], Kim, S. H.., 2008 [44]
Quality of Life Index - Cancer Version (QLI-CV).This 33-item questionnaire consists of four subscales: health and functioning, socioeconomic, psychological/spiritual, and family [58]	3	Farren, A., 2010 [51], Sammarco, A., 2008 [32], Pedro L.W.,2001 [39]
Functional Assessment of Cancer Therapy - General (FACT-G).This 27-item questionnaire has four primary QoL domains covering physical, emotional, social/family and functional well-being [21]	2	Begovic-Juhant, A., 2012 [42], Simone S.M.H., 2013 [31]
Ladder of Life provides a global single-item QOL score. Respondents are shown a stepladder with rungs from 1 to 10, where 1 represents the worst possible life and 10 represents the best possible life, and asked to circle the number that represents how they feel at the present time [59]	2	Avis N.E. 2005 [41], Ganz P.A., 2003 [52]
Quality of Life Measurement (QoL-M) assesses physical, psychological, and social aspects of adaptation to breast cancer. The tool consists of 28 items placed on 10 cm linear analog scales to measure the perceived degree of disruption related to specific side effects and outcomes of breast cancer treatment. The items assess outcomes related to emotion regulation, problem regulation, and general QOL [46]	1	Kessler, T., 2002 [46]
Quality of Life in Adult Cancer Survivors (QLACS). This 47-items questionnaire consists of 12 domains. 7 are considered generic and 5 cancer-specific. Generic domains include: physical pain, negative feelings, positive feelings, cognitive problems, sexual problems, social avoidance, and fatigue. Cancer-specific domains include financial problems resulting from cancer, distress about family, distress about recurrence, appearance concerns, and benefits of cancer [60]	1	Carver, C. S., 2006 [13]
Quality of Life Cancer Survivor Version (QOL-CS). This 41-items questionnaire consists of four QoL domains incorporating physical, psychological, social, and spiritual well-being [61]	1	Cheng, H., 2013 [34]
Global Life Satisfaction Scale (GLSS) - adapted from the Ladder of Life - asks individuals to respond to their global life satisfaction on each of three ladders for “right now,” “in 5 years,” and “compared to most people”. The ladder is a vertical, self-anchoring scale with 10 rungs. Scaling responses range from 0 (worst possible life) to 10 (best possible life) [59]	1	Kessler, T., 2002 [46]

### Frequency of psychosocial determinants measurement tools per individual QoL measures in breast cancer survivors

A range of different measurement tools were used per psychosocial determinant (Table 4). There were 10 different measures of social support used in 14 studies, with the Medical Outcomes Survey (MOS) Social Support Survey and the Interpersonal Support Evaluation List (ISEL) used most frequently (3 studies respectively). The majority of measurement tools were used in only 1 or 2 studies. Table 5 presents the frequency of the individual psychosocial determinant measures per individual QoL measure. The 14 studies that measured the influence of the determinant social support employed 10 different measures of social support and 7 different measures of QoL. The MOS Social Support Survey was used to measure social support in 3 different studies but only 2 of these studies used the same QoL measure [23, 33]. Similarly depression was measured by 4 different measures and its influence was assessed using 4 different QoL measures. While future appraisal and perspective was measured in 7 studies using 8 different measures and 6 different QoL measures; only 2 studies used the same measure for the determinant (uncertainty in illness) and QoL [32, 51].

**Table 4:** Description and frequency of psychosocial measures

Psychosocial measure	Frequency used	Primary Author, Year
***Social Support***		
Medical Outcomes Study (MOS) Social Support Survey-(19 items)- measures perceived availability of support: emotional/informational support, tangible support, affectionate support, and positive social interaction [62]	3	Ashing –Giwa K.T.. 2010 [33], Dura-Ferrandis E, 2016 [28], Goyal N. G, 2018 [23]
Interpersonal Support Evaluation List (ISEL)- 40 items- evaluates the perceived availability of four dimensions of social support consisting of belonging, appraisal, tangible and self-esteem support. Belonging support is the availability of people with whom one can do things. Appraisal support is the availability to talk to or behaviours of a supporting person, including empathy, caring, love and trust. Tangible support is instrumental aid and comprises providing support in a physical way that assists an individual in meeting their role responsibilities. Self-esteem support is the availability of a positive comparison when comparing oneself with others [63]	3	Carver C.S. 2006 [13], Huang C.Y. 2013 [36] Lewis, J., 2001 [37]
Social Support Questionnaire (SSQ-6) -6 questions asking about the affective aspects of social support. Each question has two parts, which are the number of support persons (SSQ6-N) and the satisfaction with social support (SSQ6-S) [64].	1	Cheng H, 2013 [34]
Social Networks Index - assesses participation in 12 types of social relationships. These include relationships with a spouse, parents, parents-in-law, children, other close family members, close neighbours, friends, workmates, schoolmates, fellow volunteers, members of groups without religious affiliation, and religious groups. One point is assigned for each type of relationship (possible score of 12) for which respondents indicate that they speak (in person or on the phone) to persons in that relationship at least once every 2 weeks [65].	1	DiSipio T, 2009 [35]
Emotional support from health care providers, family members and friends/co-workers (rated low vs. high). Satisfaction with partner scale was combined with marital status as follows: (1) respondent did not report a partner; (2) respondent is slightly satisfied or very satisfied with partner relationship, or (3) respondent is neutral, slightly, or very unsatisfied with their partner.	1	Janz N.K., 2014 [26]
Social Support Questionnaire (SSQ-8) is a self-administered measure of social support from five sources: spouse, family member, friend, nurse, and physician (40 items in all) [66].	1	Sammarco, A., 2008 [32]
Family APGAR- family functioning- a 5-item scale assesses participants’ satisfaction with their family’s ability to communicate, assist one another, and respond to change. Each item (e.g., “I am satisfied when I can turn to my family for help when something is troubling me”) is rated on a scale from 1 (never) to 5 (always) [67].	1	Northouse, L.L (1999) [38]
Norbeck Social Support Questionnaire (NSSQ) is a self-administered 9-item instrument that measures the multiple dimensions of social support including functional components of affirmation, aid, and affection; social network properties of frequency of contact, number in network, and duration of relationships; and recent losses of supportive relationships [68].	1	Pedro L.W. (2001) [39]
Supportive Care Needs Survey- Short Form- is a standardised instrument for measuring cancer patients’ perceived needs across a range of domains. A total number of 34-items are divided into five domains: physical/daily living (5 items), psychological (10 items), sexuality (3 items), patient care and support (5 items) and health system and information needs (10 items) [69]**.** Supportive Care Needs Survey – Health system and information needs domain only [69]**.**	2	Edib Z (2016) [40] DiSipio T., 2009 [35]
Cancer Rehabilitation Evaluation System (CARES)- a multidimensional self-administered instrument containing multiple problem areas. Administered the marital and sexual scales and the body image subscale. The marital scale includes five subscales (communication with partner, affection with partner, interaction with partner, neglect by partner and overprotection). The sexual scale included two subscales: sexual interest and sexual dysfunction [70]**.**	1	Avis N.E. (2005) [41]
***Depression***		
Centre for Epidemiological Studies-Depression (CES-D) is a screening tool for depressive symptoms and includes 20-item to investigate perceived mood and level of functioning within the past 7 days. Scores of 16 or higher are considered indicative of depression [71]**.**	2	Begovic-Juhant, A., 2012 [42], DeShields, T., 2006 [24]
Hospital Anxiety and Depression Scale (HADS) is a validated screening instrument for anxiety and depression in somatically ill patients. A score of 0 to 7 is categorized as normal, a score of 8 to 10 is considered to indicate a possible anxiety or depressive disorder, and a score of 11 or above is considered to indicate a probable anxiety or depressive disorder [72]**.**	2	Cheng A.S.K., 2016 [43], Simone, S.M. H, 2013 [73]
Becks Depression Inventory- a 21-item, self-report rating inventory that measures characteristic attitudes and symptoms of depression [74]**.**	2	Goyal N. G, 2018 [23], Kim, S.H.., 2008 [44]
Depression history -no history, history of depression without current symptoms, history of depression with current symptoms	1	Janz N.K., 2014 [26]
***Anxiety***		
Hospital Anxiety and Depression Scale (HADS) is a validated screening instrument for anxiety and depression in somatically ill patients. A score of 0 to 7 is categorized as normal, a score of 8 to 10 is considered to indicate a possible anxiety or depressive disorder, and a score of 11 or above is considered to indicate a probable anxiety or depressive disorder [72]**.**	3	Akechi, T., 2015 [45], Cheng A.S.K., 2016 [43], Simone, S.M. H, 2013 [73]
**Positive and negative affect**		
Positive and Negative Affect Scale (PANAS) is a 20-item tool that contains two 10-item scales, one measuring positive affect and the other negative affect. Positive affect items reflect the extent to which an individual feels enthusiastic, active, and alert. Negative affect items reflect subjective feelings of distress, including anger, contempt, guilt, fear, and nervousness [75]**.**	1	Kessler, T. A. 2002 [46]
**Coping**		
Brief COPE- 9 of the original 14 subscales were grouped. Active coping, instrumental support, emotional support, acceptance, and positive reframing were combined to assess Engagement Coping. The behavioral disengagement and denial subscales were combined as Disengagement Coping. Venting and self-distraction were considered as separate subscales [76]**.**	1	Dura-Ferrandis E, 2016 [28]
Brief COPE- The 28-item scale was used to measure 14 types of coping strategies. Two domains were formed from seven strategies: active coping (active coping, use of emotional support, use of instrumental support, and positive reframing) and passive coping (self-blame, denial, and behavioral disengagement) [76]**.**	2	Goyal N. G, 2018 [23], Paek, M., 2016 [25]
Family Crisis Oriented Personal Evaluation Scale (F-COPES)- assesses a family’s problem-solving strategies in response to family problems/difficulties and includes 3 external (use of outside resources) and 2 internal family coping strategies (utilize the family’s internal strengths/resources). This study focused on the following coping: external (6-item acquiring social support from friends/relatives, 3-item acquiring social support from neighbors, and 4-item seeking spiritual support) and internal (8-item reframing) family coping strategies [77]**.**	1	Paek M, 2016 [47]
Ways of Coping-Cancer Version - participants were asked to indicate how often they had used each of the following strategies in the last 6 months in attempting to cope with the most stressful part of their breast cancer; seeking and using social support, keeping feelings to self, using positive cognitive restructuring, using wishful thinking, making changes, spirituality and detachment. Three items assessed feelings of preparedness for coping with breast cancer, how well prepared patients were for the impact of cancer on their relationships, how they might feel about their appearance after surgery, and availability of counseling or support groups [78]**.**	1	Avis N.E. (2005) [41]
**Confidence and self-efficacy**		
Question- confidence about remaining cancer free- ‘To what extent do you believe that you will remain free of cancer in the future?’ answered on a nine-point scale, with 9 = absolutely sure I won’t get cancer again, 5 = I don’t know and 1 = not at all confident, I expect to get cancer again	1	Carver C.S. et al. 2006 [13]
The Self Rated Abilities for Health Practices Scale (SRAHP) is a 28-item, 5-point scale to measure self-perceived ability to implement health-promoting behaviors. SRAHP contains four subscales: Exercise, Nutrition, Responsible Health Practice, and Psychological Well Being. Each subscale has seven items. Respondents are asked to rate the extent to which they are able to perform health practices related to these four domains [79]**.**	1	DiSipio T et al., 2009 [35]
Rosenberg Self-Esteem Scale- a self-administered, 10-item scale that measures the self-acceptance aspect of self-esteem or the overall sense of being capable, worthwhile, and competent [80]**.**	1	Pedro L.W. (2001) [39]
Rosenbaum Self-Control Schedule- used as a measure of learned resourcefulness (36 items). For each of the 36 items participants indicate the degree to which it describes their behavior on a 6-point scale ranging from extremely descriptive (+ 3) to extremely non-descriptive (− 3) [81]**.**	1	Pedro L.W. (2001) [39]
**Spirituality**		
Functional Assessment of Chronic Illness Therapy - Spiritual Well-Being (FACIT-SP-12). This 12-item questionnaire consists of two subscales, one measuring a sense of meaning and peace and the other assessing the role of faith in illness [82]**.**	1	Goyal N. G, 2018 [23]
Religious Coping (RCOPE) - spiritual struggle was measured using the 7-item Negative Coping subscale of RCOPE. Examples of items on the Negative Coping subscale include “I wondered what I did for God to punish me” and “I wondered whether God had abandoned me”. Measured on a 4-point Likert-type scale ranging from 0 (not at all) to 3 (a great deal), the participants were asked to indicate the degree to which they used each strategy [83]**.**	1	Manning-Walsh J, 2005 [48]
System of Beliefs Inventory– (SBI-15R) - which measures both religious and spiritual aspects of belief systems in coping with a life-threatening illness. The SBI-15R encompasses both constructs by assessing beliefs and practices of faith systems (10 items, Subscale I) and social support from the religious and/or spiritual community (5 items, Subscale II), and applies equally to atheists, agnostics, those with no religious affiliation, and those with a moderate or strong religious or spiritual orientation [84]**.** The SBI-15R was modified to include 4 items from the Beliefs and Practices subscale (e.g. “Religion is important in my day-to-day life”, “Prayer has helped me cope during times of serious illness”) and four items from the Social Support subscale (e.g., “I enjoy attending religious functions held by my religious or spiritual group”, “I know someone in my religious or spiritual community that I can turn to”). Items from each subscale were averaged for all participants, with a range of values from 1 (strongly disagree) to 5 (strongly agree) [84]**.**	2	Wildes, K.A. 2009 [49] Janz N.K., 2014 [26]
**Optimism**		
Life Orientation Test- comprised of 8 items, plus 4 filler items that are not calculated in the total score. Each item (e.g., “I always look on the bright side of things”), is rated on scale from 0 (strongly disagree) to 4 (strongly agree). Individual items are summed (excluding the filler items) with higher scores indicating more dispositional optimism [85]**.**	4	Carver C.S. et al. 2006 [13], Dura-Ferrandis E, 2016 [28], Goyal N. G, 2018 [23], Northouse, L.L (1999) [38]
The Minnesota Multiphasic Personality Inventory (MMPI) – The original MMPI is a 566-item self-report inventory that utilizes a true/false response format. The MMPI yields information about personality factors related to psychiatric syndromes. The optimism-pessimism (PSM) scale was developed using 298 MMPI items [86]**.**	1	Petersen L.R. (2008) [50]
**Future perspectives and appraisal**		
Mishel Uncertainty in Illness Scale – Community Form (MUISC) is a 23-item, 5-point (strongly disagree to strongly agree), Likert-type scale, self-administered measure of the uncertainty perceived in illness [87].	2	Sammarco, A., 2008 [32], Farren, A. T, 2010 [51]
Appraisal of illness scale- consists of 27 scored items (e.g., “this situation threatens to overwhelm me”) and 5 unscored filler items, with a 5-point Likert-type response format with choices ranging from 1 (very false) to 5 (very true) [88]**.**	1	Northouse, L.L (1999) [38]
Power as Knowing Participation in Change Tool (PKPCT) is a 52 item semantic differential scale designed to measure an individual’s capacity to participate knowingly in change on four subscales (awareness, choices, freedom to act intentionally, and involvement in creating change) [89]**.**	1	Farren, A. T, 2010 [51]
Self-Transcendence Scale (STS) measures the capacity for self-transcendence. It is a unidimensional, 15-item, 4-point Likert scale. The scale ranges from not at all to very much [90]**.**	1	Farren, A. T, 2010 [51]
Received enough information from their doctors or the staff about risk of breast cancer recurrence (yes/no). (Time 1) Perceived likelihood of breast cancer recurrence (“not at all likely” to “very likely to recur”). (Time 2) Worry about recurrence-concern the cancer would recur in the same breast, the other breast, or to another part of the body. (Change from Time 1 to Time 2 scores Decision regret -categorized as a lot of decision regret versus none or some decision regret [91].	1	Janz N.K., 2014 [26]
Fear of Progression Questionnaire-Short Form- (FoP-Q-SF) consists of 12 items pertaining to four scales (affective reactions, partnership/family, occupation, and loss of autonomy), answered on a Likert scale (frequency of experience of fear/worry: 1 = never to 5 = very often) [92]**.**	1	Koch, L 2014 [29]
Concerns of Recurrence Scale (CARS) assesses the extent and the nature of women’s concerns about breast cancer recurrence. Two components; *Overall fear index* includes 4 questions on frequency, potential for upset, consistency, and intensity of fears. Scores are given on a six-point Likert scale that ranges from 1 (not at all) to 6 (continuously). *The nature of women’s fears about recurrence* includes 26 items subdivided into four domains: health worries, womanhood worries, role worries, and death worries. Health worries (11 items) measures concern about future treatment (e.g., chemotherapy, radiation, and surgery), emotional upset, physical health, carrying out planned activities, and loss of breast(s). Womanhood worries (7 items) measure femininity, sexuality, womanhood, body image, romantic relationships, identity, and spirituality or faith. Role worries (6 items) measure roles and responsibilities at work and at home, relationships with friends and family, physical ability to complete daily activities, financial problems, and self-confidence. Death worries (2 items) measure the possibility that a recurrence of breast cancer could lead to death. Scores range from 0 (not at all), 1 (a little), 2 (moderately), 3 (a lot), to 4 (extremely), to indicate the extent to which they worry about each item [93]**.**	1	Taylor, T.R., 2012 [30]
Perceptions of life- a 12-item scale to measure perceptions of life after cancer (developed by the authors). Example items include “Surviving breast cancer has changed my outlook on life,”, “I get less worried about trivial things,” and “I feel more vulnerable now, as if the world is a more dangerous place.” Respondents indicate the extent to which they believe their outlook has changed on a 5-point scale, ranging from 0 (not at all) to 4 (very much). The first factor includes six items assessing changes in perspectives and priorities as a measure of positive meaning. The second factor includes five items assessing fears about recurrence and about one’s body, and measures vulnerability [94]**.**	1	Ganz P.A. (2003) [52]
**Impact of Events**		
Impact of Events Survey (IES)- The IES is a 15 item self-report measure of intrusive thoughts and avoidance associated with a stressor (breast cancer). In this study the Intrusion subscale of the IES is considered a measure of processing. Participants rated how true each statement has been for them in the past 3 weeks, using the following scale: 0 = Not at all, 1 = Rarely,3 = Sometimes, and 5 = Often. All statements were anchored to the participant’s cancer and its treatment, such as “Thought about it when I didn’t mean to” and “I had dreams about it.” [95]	1	Lewis J., 2001 [37]
Illness Intrusiveness Rating Scale- measures the impact of cancer on multiple life areas (13-items). Using a Likert scale ranging from one (not very much) to seven (very much), participants rate the degree of interference caused by their illness or its treatment with 13 aspects of their lives. These domains are: health, diet, work, active and passive recreation, financial situation, relationship with spouse, sex life, family and other social relations, self-expression/self-improvement, religious expression and community/civic involvement [96]**.**	1	Goyal N. G, 2018 [23]
Impact of Cancer scale–is a self-report instrument that is designed to capture how long-term survivors interpret the overall positive and negative impacts of having cancer in their lives. Item responses are in a five-point Likert scale format where respondents are asked to give their overall agreement from 1 (strongly disagree) to 5 (strongly agree). Mean scores are compiled for each domain and then aggregated into the two meta-domains: the positive impact of cancer (PIC) and the negative impact of cancer (NIC) [97]**.**	1	Bouskill K. (2016) [53]
***Stress***		
Post-traumatic Stress Disorder Checklist Civilian Version (PCL-C) -assesses post-traumatic stress symptoms. The PCL-C is a 17-item self-report checklist of PTSD symptoms based closely on the DSM-IV criteria. Respondents rate each item from 1 (“not at all”) to 5 (“extremely”) to indicate the degree to which they have been bothered by that particular symptom over the past month [98]**.**	1	Morrill, E.F., 2008 [54]
Omega Screening Questionnaire (OSQ) - is comprised of four parts: (a) demographic and background information, (b) health history, (c) inventory of current concerns, and (d) symptoms scale. The demographic section of the OSQ includes a number of questions about the respondent’s age, education, income, and so forth. The Inventory of Current Concerns is a 40-item scale that asks participants to rate the extent to which they have experienced a list of concerns about issues such as finances, children and work in the past month. Participants rate each item according to whether the statement is not true (0), somewhat true (1), or true (2) for them. The Symptoms Scale asks participants to rate the extent to which they have experienced 13 symptoms (e.g., fatigue, breathing problems, pain). Response options are 0 (no trouble), 1 (some), and 2 (a lot) [99]**.**	1	Northouse, L.L (1999) [38]
Life Stress Scale – Life burden- which assesses the level of stress associated with various aspects of daily living. Scale consists of family (6-items), functional (4-items), and neighborhood stresses (6-items), Items are rated from 1 to 5, with a higher score indicating less life burden/ stress, and calculated into a mean score [100]**.**	1	Ashing –Giwa K.T.. 2010 [33]
Amount of stress (very little, some, a moderate amount, a lot) Perceived handling of stress (not well at all, not well, fairly well, very well)	1	DiSipio T et al., 2009 [35]
FACT-B Additional Concerns subscale. Negative self-image was measured using two items (e.g. “I feel sexually attractive” and “I am able to feel like a woman”) [21]	1	Paek M, 2016 [47]
Urban Life Stress Scale assesses the level of life-related stress for the past 3-month [101]**.** In this study, a three-factor structure was selected and named as “functional stress” (e.g., finances, job situation; 3-item), “stressful life-events” (e.g., illness of someone close; 2-item), and “role stress” (e.g., parenting; 3-item).	1	Paek M, 2016 [47]
The Family Communication Scale of the Family Adaptability and Cohesion Evaluation (FACES-IV) [102] and the Family Avoidance of Communication about Cancer (FACC) Scales [103] were used to assess both general and cancer-specific family communication problems. A composite score was created by averaging the z scores of both measures, with greater scores representing higher communication strain.	1	Paek M, 2016 [47]
**Posttraumatic Growth**		
Posttraumatic Growth Inventory-an instrument for assessing positive outcomes reported by persons who have experienced traumatic events. This 21-item scale includes factors of New Possibilities, Relating to Others, Personal Strength, Spiritual Change, and Appreciation of Life [104]	2	Morrill, E.F., 2008 [54], Bellizzi K.M. (2009) [27]
**Cognitive Symptoms**		
Cognitive Symptoms Checklist-Work-21 items are used to assess work-related cognitive problems. The original English version consists of three subscales, including working memory, executive functioning, and attention. The Chinese version used by this current study applied a two-factor instead of three-factor structure that combined items measuring task completion and executive function [105]**.**	1	Cheng A.S.K., 2016 [43]
**Work Limitations**		
Work Limitation Questionnaire (WLQ); measures the degree of work limitation, which is inversely related to work productivity. The 25-item WLQ consists of four subscales: time demands, physical demands, mental interpersonal demands, and output demands; and users rate their ability or level of difficulty in fulfilling the job demands on a scale ranging from 1 to 5 [106]**.**	1	Cheng A.S.K., 2016 [43]
**Healthcare System**		
Health care system- patient-doctor relationship (6 items), comfort in health care system (3 items) and diagnostic care delay (1 item). Patient–doctor relationship was assessed from the Interpersonal Aspects of Care subscale of the Adherence Determinants Questionnaire. This measure focuses on interpersonal aspects of care, communication, and rapport on a scale from 1 (strongly disagree) to 5 (strongly agree). (113) Comfort in health care system included use of regular medical check-ups, comfort using the health care system, and comfort in asking questions. Each score was standardized ranging from 0 to 100 and averaged to obtain an overall score. Diagnostic care delay was assessed by asking respondents how long (number of days) they waited to obtain medical care from the time they first noticed something was wrong. It was calculated by the time interval (days) between the first symptom and medical diagnosis	1	Ashing –Giwa K.T.. 2010 [33]

**Table 5:** Frequency of psychosocial determinant measures per individual QoL measure

	FACT-B	SF-36	EORTC QLQ-C30	EORTC QLQ-BR23	FACT-G	QLI-CV	Ladder of Life	QOLM	QLACS	QOL-CS
**Social Support**										
Medical Outcomes Study (MOS) Social Support Survey- 3 items	2 [23, 33]	1 [33]	1 [28]							
Interpersonal Support Evaluation List (ISEL)		2 [36, 37]							1 [13]	
Social Support Questionnaire (SSQ)- 6 items										1 [34]
Social Support Questionnaire (SSQ)- 8 items						1 [32]				
Norbeck Social Support Questionnaire- 9 items						1 [39]				
Social Networks Index	1 [35]									
Supportive Care Needs Survey	1 [35]		1 [56]							
Cancer Rehabilitation Evaluation System (CARES)	1 [41]						1 [41]			
Emotional support from others and satisfaction with partner scale	1 [26]									
Family APGAR-family functioning	1 [38]									
**Depression**										
Center of Epidemiologic Studies Depression Scale (CES-D)	1 [24]			1 [42]	1 [42]					
Hospital Anxiety and Depression Scale (HADS)			1 [43]		1 [73]					
Becks Depression Inventory	1 [23]		1 [44]	1 [44]						
Depression history	1 [26]									
**Anxiety**										
Hospital Anxiety and Depression Scale (HADS)			2 [43, 45]		1 [73]					
**Positive and negative affect**										
Positive and Negative Affect Scale (PANAS)								1 [46]		
**Coping**										
Brief COPE- Engagement Coping, Disengagement Coping, Venting and Self-Distraction			1 [28]							
Brief COPE- active/positive coping and passive/negative coping	2 [23, 25]									
Family Crisis Oriented Personal Evaluation Scale (F-COPES)	1 [47]	1 [47]								
Ways of Coping-Preparedness	1 [41]						1 [41]			
**Confidence and self-efficacy**										
Question- confidence about remaining cancer free									1 [13]	
Health efficacy	1 [35]									
Rosenberg Self-Esteem Scale						1 [39]				
Rosenbaum Self-Control Schedule						1 [39]				
**Spirituality**										
Functional Assessment of Chronic Illness Therapy- Spiritual Well-being Scale	1 [23]									
Religious Coping- negative coping subscale	1 [48]									
Systems of Belief Inventory	2 [26, 49]									
**Optimism**										
Life Orientation Test	2 [23, 38]		1 [28]						1 [13]	
The Minnesota Multiphasic Personality Inventory (MMPI) – optimism-pessimism (PSM) scale		1 [50]								
**Future perspectives and appraisal**										
Mishel Uncertainty in Illness Scale						2 [32, 51]				
Appraisal of illness	1 [38]									
Power as Knowing Participation in Change Tool						1 [51]				
Self-Transcendence Scale						1 [51]				
Recurrence information, likelihood and worry about recurrence, decision regret	1 [26]									
Fear of Progression Questionnaire			1 [29]	1 [29]						
Concerns of Recurrence Scale	1 [30]									
Vulnerability- fears about recurrence		1 [52]					1 [52]			
**Impact of Events**										
Impact of Events Survey		1 [37]								
Illness Intrusiveness Rating Scale	1 [23]									
Impact of Cancer		1 [53]								
**Stress**										
Post-traumatic Stress Disorder Checklist	1 [54]									
Omega Screening Questionnaire	1 [38]									
Life Stress Scale	1 [33]	1 [33]								
Amount of stress and perceived handling of stress	1 [35]									
FACT-B additional concerns	1 [47]	1 [47]								
Urban Life Stress Scale	1 [47]	1 [47]								
The Family Communication Scale of the Family Adaptability and Cohesion Evaluation (FACES-IV) and the Family Avoidance of Communication about Cancer (FACC)	1 [47]	1 [47]								
**Post-traumatic Growth**										
Post-traumatic Growth Inventory	1 [54]	1 [27]								
**Cognitive Symptoms**										
Cognitive Symptoms Checklist-Work			1 [43]							
**Work Limitations**										
Work Limitations Questionnaire			1 [43]							
**Healthcare System**										
Patient-doctor relationship, comfort in healthcare system and diagnostic care delay	1 [33]	1 [33]								

*FACT-B* Functional Assessment of Cancer - Breast Cancer, *SF-36* Medical Outcomes Study Short Form, *EORTC QLQ-C30* The European Organisation of Research and Treatment of Cancer Quality of Life Questionnaire, *QLQ-BR23* The European Organisation of Research and Cancer Treatment Quality of Life Questionnaire - Breast Cancer, *FACT-G* Functional Assessment of Cancer Therapy - General, *QLI-CV* Quality of Life Index - Cancer Version, *QoL-M* Quality of Life Measurement, *QLACS* Quality of Life in Adult Cancer Survivors, *QOL-CS* Quality of Life Cancer Survivor Version, *GLSS* Global Life Satisfaction Scale

### Assessing the influence of the psychosocial determinants on QoL in breast cancer survivors

Among the studies that investigated social support, the general conclusion was that low perceived support was associated with a worse QoL [35, 41, 56] and higher levels of support were associated with better QoL [13, 23, 28, 32, 34, 37, 38]. Three studies found that the influence of social support on QoL varied by the type of support e.g. appraisal, belonging [36, 39]. Only 2 studies found no association between social support and QoL [26, 33].

For depression, 6 studies showed an inverse relationship with higher/lower levels of depression associated with a lower/higher QoL [23, 24, 26, 31, 42, 44] and one study found no association [43]. Out of the 7 studies that investigated future appraisal and perspectives, fear/worry about cancer recurrence was associated with a lower QoL in 4 studies [26, 29, 30, 52]. Higher uncertainty about illness was found to be associated with a lower Qol in 2 studies [32, 51] and appraisal of illness was also shown to mediate the influence of concerns and optimism on women’s QoL in one study [38].

The 5 studies that looked into coping generally found that the most relevant aspect of coping was the type of coping strategy one used, with disengagement, self-distraction, keeping to self and wishful thinking all having a negative association with QoL [28, 41, 47]. In general higher utilisation of active coping and lower utilisation of passive coping were positively associated with QoL [23, 25, 41]. Four of the 5 studies that assessed the role of optimism on QoL found that higher levels of optimism were positively associated with QoL [13, 23, 28, 50]; the remaining study was null [38]. All 5 studies that investigated stress found that greater psychological stress, symptom distress and life burden were associated with a lower QoL [33, 35, 38, 47, 54].

The 4 studies that examined the association between faith or spirituality and QoL had mixed findings. One study found that women with greater spiritual beliefs were more likely to have a lower emotional QoL [26], and another study reported that spiritual struggles were associated with lower QoL [48]. The other 2 studies concluded that higher rates of engagement with faith and spirituality had a positive impact on QoL [23, 49].

For the 3 studies that assessed anxiety, 2 studies reported that higher anxiety was associated with a lower QoL [31, 45] while the remaining study found a null association [43]. In 3 individual studies higher confidence, self-efficacy and self-esteem were each found to be associated with higher QoL [13, 35, 39]. Higher frequency of intrusive thoughts in 2 studies [23, 37] and a perceived negative impact of cancer in 1 study were associated with a lower QoL [53].

Two studies found that higher scores for posttraumatic growth were associated with a higher QoL [27, 54]. While higher scores on negative affect and cognitive limitations were found to be associated with a lower QoL [43, 46]. Work limitations were reported to have no significant impact on QoL [43]. The one study on health care system determinants found that a positive patient-doctor relationship was associated with better psychological well-being, while diagnostic care delay was associated with lower physical well-being in some ethnic groups [33].

## Discussion

This review confirms that there are numerous psychosocial determinants that are associated with QoL in breast cancer survivors. The psychosocial determinants investigated most frequently were social support, depression and future appraisal and perspective. There was less research undertaken on societal determinants, such as healthcare system factors, work limitations etc. In general, across all the 33 articles included in this review, a higher level of a positive influence and a lower level of a negative influence of a psychosocial determinant was associated with a better QoL e.g. higher social support and lower levels of depression were found to be associated with a higher/better QoL. There were some determinants such as spirituality and coping were the influence on QoL was mixed or it varied, depending on which aspect of the determinant was measured e.g. type of coping strategy; but these determinants were also less commonly investigated.

This review also identified a range of gaps and limitations in the current literature and areas for further research. The majority of studies were cross-sectional and assessed the influence of psychosocial determinants on QoL at a single point in time. It is possible that the influence of psychosocial determinants on QoL may vary over time. A US study of breast cancer survivors found that when worry about recurrence increased over time (4 years after diagnosis), women were more likely to report a decline in emotional well-being. On average, there was a gradual lessening of worry as the years of survivorship increased, but some women reported greater worry at 4 years than they did shortly after primary treatment was completed [26]. The majority of the studies were undertaken in North America and the findings may not be transferable to other countries, with differing health care systems and cultures.

There is also considerable variation in the type of measures being used to assess both QoL and the individual psychosocial determinants across studies. There were 12 different QoL measures utilised across the 33 studies. Some of the QoL measures were breast cancer specific (FACT-B), some were cancer focussed (though not specific to a particular cancer; EORTC QLQ-C30) and some were generic (SF36) and hence may not have focussed on the same aspects of QoL aspects. Thus findings may not be comparable. A systematic review of QoL instruments in long-term breast cancer survivors identified only three instruments (QLACS, QLI-CV, QOL-CS) that evaluated all four domains of QoL (physical, psychological, social and spiritual) [107]. These instruments were only used in 5 studies in the current review (Table 3). Similarly, this review identified that on average 6 different measures were used per psychosocial determinant, making comparability of findings difficult.

While the findings provide evidence of a relationship between individual psychosocial determinants and QoL, they are not conclusive. Across the 34 studies there was only ever a maximum of 2 studies where results could be directly compared and this was only feasible for 6 determinants; social support, anxiety, coping, spirituality, optimism and future perspectives and appraisal. (Table 5) The clinical relevance of the possible effects of the determinants on QoL is also difficult to interpret. Differences in QoL should be compared to the minimal important difference for the various QoL measures, if known e.g. estimated to be in the range of 3–8 points for the FACT-B [108, 109]. It is also possible given the breadth in definition of a “psychosocial determinant”, that there are a range of other determinants whose influence on QoL has yet to be measured in studies e.g. motivation, goals. A recent systematic review identified that cancer may impact patients’ life goals and life goal disturbance may be related to poorer psychological outcomes but further studies are required [110].

This is the first scoping review of the psychosocial determinants of QoL in breast cancer survivors. However there were some limitations to this review process. It is feasible that despite an extensive search of multiple databases, some relevant papers may have been missed. Not all abstracts were screened by two independent reviewers; 75% were screened. However the adaption of the inclusion/exclusion criteria by the two independent reviewers as part of the scoping review iterative process allowed for a more focused review by alleviating any potential ambiguity, given the broad research question [20]. There was also no quality appraisal or meta-analysis of the included studies undertaken, but again is not deemed to be part of the scoping review process [19].

Recently there has been an emphasis on developing more patient-centered care in breast cancer survivors and using an individual’s psychological needs as a guide for psychosocial treatment selection rather than their diagnostic or medical treatment [111]. Understanding the influence of psychosocial determinants on QoL in breast cancer survivors potentially helps to enable the development of more personalised and tailored intervention strategies and support services to reduce long term physical and psychological morbidity. The identified psychosocial determinants can be mapped to evidence based psychosocial treatments such as Cognitive and Behavioral Cancer Stress Management to provide patients with skills to live well with breast cancer and/or improve QoL [112].

## Conclusion

This review has identified several psychosocial determinants of QoL in breast cancer survivors. The overall consistency of the associations found between the various psychosocial variables and QoL, regardless of the measures used, provides a reasonably clear picture of the influence of individual psychosocial determinants on QoL in breast cancer survivors. The fact that these associations do not depend on the specific measures used adds validity to the findings. However this review has also highlighted a clear need to standardise measures of both QoL and individual psychosocial determinants, potentially through expert consensus groups, in order to be able to evaluate the impact of psychosocial determinants on QoL systematically and to compare results across studies. Further research also needs to be undertaken in health care settings, outside of the USA; given that psychosocial determinants and QoL itself may in fact be influenced by the organisation and availability of follow-up clinical and supportive care. Future studies should also use a prospective or longitudinal design to monitor change and understand the complexity and variety of influences on QoL long-term. By improving the quality of evidence on this topic there is the potential to also improve the quality of follow-up care in breast cancer survivors.

## Data Availability

Not applicable

## Appendix

**Table 6:** Database search criteria

**PubMed**			
Survivors(MeSH Terms) OR survivor*(Title/Abstract)	breast neoplasms (MeSH Terms) OR Breast cancer(Title/Abstract)	QOL (MeSH Terms) OR HRQOL OR physical OR emotional OR functional OR social	women OR female
**EMBASE**			
‘survivors’/exp. OR survivor*:to,ab	‘breast cancer’/exp. OR ((breast NEXT/1 cancer):ti,ab) OR ((breast NEXT/1 neoplasm*):ti,ab)	QOL OR HRQOL OR physical OR emotional OR functional OR social	women OR female
**PsycINFO**			
DE “Survivors+” OR survivor*	(DE “Breast Neoplasms+”) OR breast N1 cancer	QOL OR HRQOL OR Physical OR Emotional OR Functional OR Social	women OR female
**CINAHL**			
MH “Survivors+” OR survivor*	(MH “Breast Neoplasms+”) OR breast N1 cancer	QOL OR HRQOL OR Physical OR Emotional OR Functional OR Social	women OR female

## Abbreviations

QoL: Quality-of-life
FACT-B: Functional Assessment of Cancer - Breast Cancer
FACT-G: Functional Assessment of Cancer Therapy – General
SF-36: Medical Outcomes Study Short Form
EORTC QLQ-C30: The European Organisation of Research and Treatment of Cancer Quality of Life Questionnaire
QLQ-BR23: The European Organisation of Research and Cancer Treatment Quality of Life Questionnaire - Breast Cancer
QLI-CV: Quality of Life Index - Cancer Version
QoL-M: Quality of Life Measurement
QLACS: Quality of Life in Adult Cancer Survivors
QOL-CS: Quality of Life Cancer Survivor Version
GLSS: Global Life Satisfaction Scale
